# Subtle temperature increase can interact with individual size and social context in shaping phenotypic traits of a coldwater fish

**DOI:** 10.1371/journal.pone.0213061

**Published:** 2019-03-27

**Authors:** C. A. Leblanc, K. Horri, S. Skúlason, D. Benhaim

**Affiliations:** 1 Department of Aquaculture and Fish Biology, Hólar University College, Saudárkrókur, Iceland; 2 Ifremer, Laboratoire Ressources Halieutiques, Centre Manche Mer du Nord, Boulogne-sur-Mer, France; 3 UMR-I 02 SEBIO, INERIS, URCA, ULH, Unité Stress Environnementaux et BIOsurveillance des milieux aquatiques, FR CNRS 3730 Scale, Université Le Havre Normandie, Le Havre, France; Uppsala Universitet, SWEDEN

## Abstract

Temperature and individual egg size have been long studied in the development of fishes because of their direct effects on individual fitness. Here we studied the combined effects of three important factors for fish development, i.e. egg size, social environment and water temperature. Arctic charr (*Salvelinus alpinus*), a coldwater fish known to be phenotypically plastic, was used to investigate how these factors may affect growth and foraging behaviour of juvenile fish in a benign environment. We accounted for the social environment during early development by comparing fish raised in groups and in isolation. We examined the effect of egg size and a 2 °C difference on foraging behaviour, activity and growth a few weeks after first feeding. Growth trajectories of fish originating from large and small eggs were similar within each temperature: larger fish coming large eggs were at all time larger than smaller fish. There was no indication that small fish raised at a higher temperature grew faster than larger fish raised at a lower temperature. A 2 °C difference in temperature affected the behaviour of fish differently according to body size and/or social context. The foraging probability difference between fish raised in groups and fish briefly isolated was higher at 4.5 °C than at 6.5 °C for both size fish. Finally, there was no repeatability in foraging behaviour and mobility for isolated individuals. These results highlight the importance of small changes in temperature when evaluating growth and behaviour of fishes, and reveal the importance of considering the interaction of temperature with other factors, e.g. individual size and social environment, especially at early stages of development in fishes. We discuss these findings in the context of rapid changes in temperature and how temperature and its interaction with other factors may affect the phenotypes, ecology and evolution of coldwater fishes.

## Introduction

In fishes, the correlation between egg size and female body size [1,2], as well as the relationship between egg size, offspring size and survival at hatching, [3,4] has been well studied. Most of these studies have been conducted on salmonids because of their large eggs (3–8 mm in diameter) and commercial value [5]. Offspring from larger eggs typically have higher fitness, e.g. higher survival, and greater resistance to starvation [6,7]. However, these effects are believed to be limited to a short period following hatching, i.e. they tend to decline rapidly throughout development, especially when fish start feeding [8]. Einum & Fleming [9] showed for brown trout (*Salmo trutta*) that when sibling groups originating from small and large eggs were reared separately in a simple non-risk (i.e. benign) environment, initial size difference disappeared rapidly. In Chinook salmon (*Oncorhynchus tshawytscha)* and steelhead trout (*Oncorhynchus mykiss*), offspring from smaller eggs often grew faster than offspring from larger eggs and were capable of catching up in size with the latter or even becoming larger [10–13]. This reduction in maternal effects during ontogeny can be partly explained by the overall environment experienced by the fish during development, and by additive genetic variance [14]. In Arctic charr (*Salvelinus alpinus*), another coldwater fish, a positive correlation between egg size and body size may last well beyond first feeding [15]. Therefore, the persistence of correlations between egg size and phenotypic traits of the offspring may be different across species and environments and may have potential implications for both juvenile and adult phenotypes.

Like other salmonids, Arctic charr show considerable variability in egg size (and yolk) within and among females, resulting in a wide size distribution of juveniles at first feeding [16–19]. The relationship between egg size and juvenile size may persist for up to one year after first feeding [20], and the relationship between juvenile and adult size may persist for up to two and a half years [15]. It has been shown that metabolic rate of Arctic charr is egg-size dependent, with smaller juveniles coming from small eggs having a higher metabolic rate than larger individuals coming from larger eggs [21]. This persistence of an egg-size effect was also seen in behavioural differences at the onset of first feeding i.e. feeding strategies and mobility in relation to body size that favour large individuals [10,22]. Therefore, behaviour and growth of individuals may be affected by both egg size and temperature, but these effects may be context dependent.

Briefly after emergence, Arctic charr and salmonid juveniles found in lakes and rivers show a wide diversity in their social environment; they have been described schooling or spatially segregated defending a territory [e.g. 23, 24]. Social environment early in life strongly influences developmental, physiological and behavioural trajectories [25,26], and can induce different evolutionary trajectories [27]. Social environments can be critical for the performance of first-feeding fish. In Arctic charr both small and large juveniles are more mobile and feed more when in the presence of conspecifics [10]. Here, egg-size effects are not cancelled out by the effect of social environment but rather interact with it to affect early behaviour and ultimately growth of individuals [10]. Specifically, larger individuals in groups are more mobile and feed more than larger fish in isolation [10]. Although the social environment of juvenile fish and early size variation are recognised as important factors in the development of behaviour, their effects in combination with other factors such as temperature are rarely examined (but see [28,29]). This is a significant weakness because temperature is a prime determinant of development in a number of fish taxa. This has been particularly well studied in salmonids.

Temperature is one of the most important environmental factors for ectotherms at all life stages, and fish can perceive temperature changes of 0.5 °C [30]. Temperature directly influences rate of development, growth [31,32], physiology and metabolism [33]. Specifically, it influences energy demands [34,35], food consumption and feeding activity [36]. For example, Whitney et al. [29] showed that elevated temperatures increased development rates, resulting in earlier hatching and shorter body length in sockeye salmon (*Oncorhynchus nerka*). Interestingly, body mass at hatching was not affected by temperature or population but rather by egg size [29], indicating that both temperature and egg size (and potentially their interaction) are important for developing embryos among and within populations of salmonids. Temperature can also affect the scaling relationships between metabolism and body size. A decrease in mass-scaling exponent was observed along an increase in temperature in Coregonids but those effects may vary among taxa [37]. Considering how much is known about the effects of temperature on physiology, its effect on behavioural traits is poorly studied in fishes (e.g. [38,39]). Most studies focus on swimming and foraging behaviour in relation to temperature (e.g. [40]). In a population, individual responses to changes in temperature may differ among juveniles. For example, juveniles that hatch in early spring may be exposed to colder temperature than juveniles emerging later. Therefore, fish emerging at different times may encounter different temperatures when they start feeding [41,42]. The onset of feeding in fishes is a critical developmental stage which may affect later food intake and growth [43,44]. Local temperature at first feeding could be a key factor in driving movement and emigration of offspring from the hatching site, which can be critical for habitat use, dispersal and migration patterns in polymorphic species like Arctic charr [23].

As described above, each of these factors can independently affect energetic requirements and therefore growth of juvenile fish, but clearly egg/body size, social context and temperature can interac and result in phenotypic variation in fishes, especially at early life stages. Often, egg size and temperature interactions have been shown to have an effect on metabolism and growth. Ecological factors such as temperature can affect metabolic rate and metabolic rate scaling with body mass within [45] and among species [46]. In Chinook salmon, the correlations between egg size and early life history traits decreased when temperature increased [47]. The temperature dependence of egg-size effect may be related to changes in the scaling of the relationship between body mass and metabolic rate that seems to be lost when applying temperature in the upper range of thermal tolerance of a species [48]. The effects of these factors may vary among species and developmental stages as well as with the magnitude of the temperature treatment. Many of the studies looking at temperature and its correlation with other factors have looked at large temperature differences (e.g. [37,47–48]), but the effect of small and stable temperature differences is still unknown, especially when looking at its effects at multiple phenotypic levels.

In summary, consequences of subtle temperature change, in line with current projections of temperature change in the Arctic and Sub-Arctic [49], on early behaviour, movement and growth of fish are largely unknown, especially as they may be combined with maternal effects such as egg size. Here we examine the interactions between two different temperatures (4.5 and 6.5 °C) and social environments (long-term and brief isolation vs. group; see also [10]) and its effect on foraging behaviour, mobility, aggressive interactions and growth of Arctic charr juveniles. We complement this study by adding a third factor of interest, the initial egg size the fish originated from (small *versus* large), as this has been shown to be important for phenotypic variation for this species [10,15,22]. In a first experiment we compare fish in groups versus fish briefly isolated (isolated for 24 hours) at both temperatures to test for the social promotion of activity and foraging. This social environment reflects conditions (schooling vs. isolated) in which juveniles can be found in lakes or rivers shortly after first feeding. First, we predicted that fish reared at a higher temperature and raised in a group would feed more, be more mobile, swim higher up in the water column, display agonistic behaviour and be larger at all times. This is based on previous work documenting behaviour and growth in similar social contexts at an average temperature between the two treatments applied in this study [22]. Second, if temperature has a large effect on both behaviour and growth of first-feeding fish, we predicted that small individuals reared at 6.5 °C and large individuals reared at 4.5 °C in groups would not differ in weight. This is based on the general assumption that temperature governs growth and metabolism of ectothermic animals as well as on previous results on growth of small and large Arctic charr [10]. If this is true this would also mean that a 2 °C increase in temperature will have an effect on metabolic rate, although we do not explicitly test for this. In a second experiment we characterised foraging and mobility of first-feeding fish that have never experienced social interaction (i.e. long-term isolation hereafter) at both temperatures and coming from small and large eggs. This may reflect a situation in the wild wherein fish leave their natal habitats quickly after emergence [24,50], and this allows for testing of the effect of temperature and size regardless of promotion of activity by social interaction [22]. Also, because we repeatedly measured the same individuals over time, we were able to quantify individual variation in behaviour as well as evaluate its consistency over time. We predicted that larger fish raised at 6.5 °C will be more mobile and feed more than small fish raised at 4.5 °C. We expected to see variation in behaviour among individuals based on body size with some consistency over time, which may suggest different personalities (although not tested in this study). Because Arctic charr in the wild shows a high level of polymorphism associated with food resources and foraging behaviour [51,52], we discuss how small differences in size at first feeding in interaction with temperature and social environment may promote differences in mobility patterns with potentially important consequences for habitat and food selection.

## Material and methods

This study was approved by the ethical committee of Hólar University College Aquaculture Research Station (HUC-ARC). HUC-ARC has an operational license according to Icelandic law on aquaculture (Law 71/2008), which includes clauses of best practices for animal care and experiments.

### Animal husbandry

**Fig 1:**
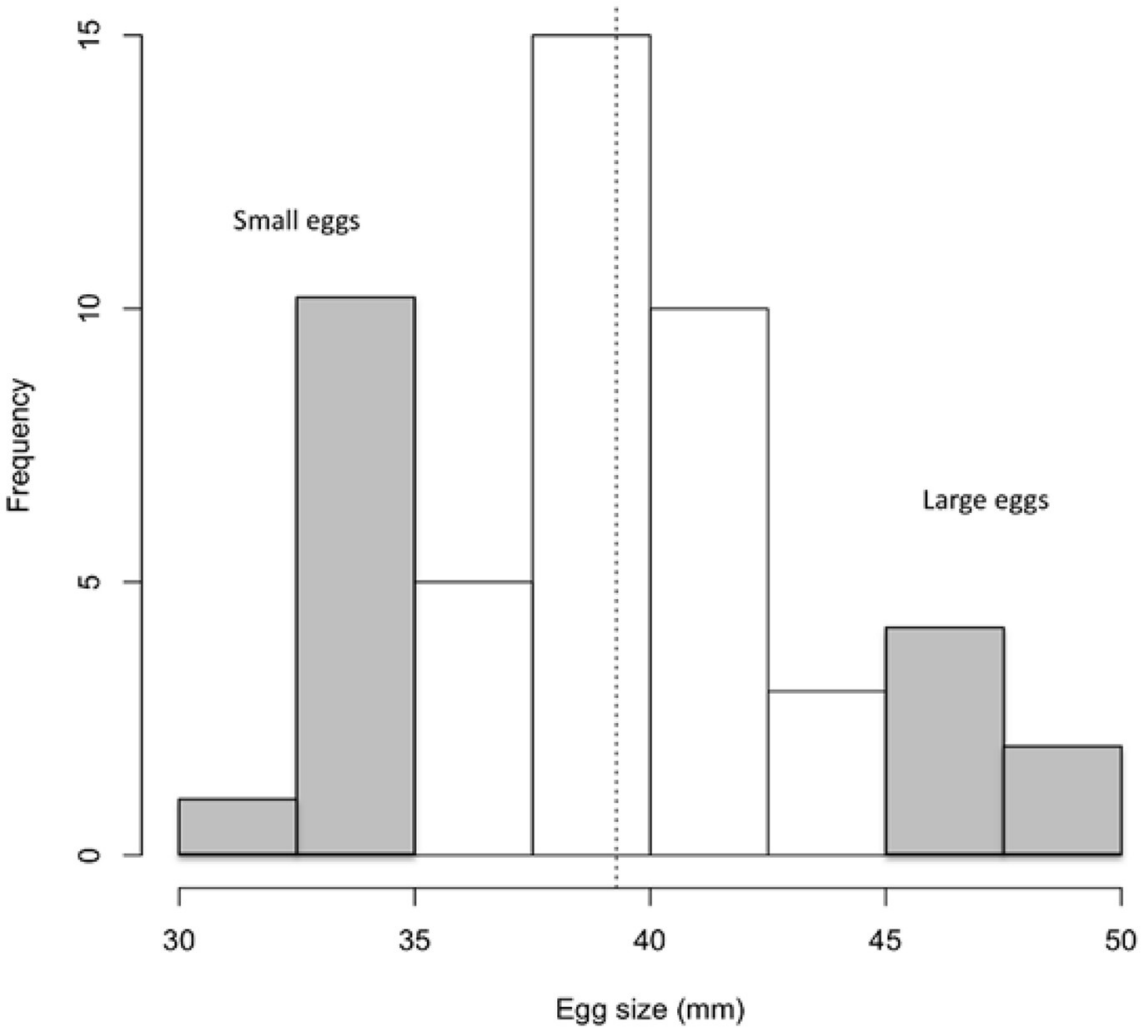
Egg size distribution of Icelandic Arctic charr (*Salvelinus alpinus*). Eggs were obtained from 15 females and four males (all age 4) from a fourth-generation breeding program at Hólar University College. The dashed line represents the mean egg size in the distribution 39.28 ± 4.72 mm (mean ± SD; median = 39 mm). The grey bars represent the larger and smaller eggs that were selected for the experiment.

Eggs were obtained from a fourth generation of Icelandic Arctic charr *S*. *alpinus* from the breeding program of Hólar University College, Iceland. Offspring used in this study came from the fertilization of a pool of 15 females with the sperm of four males (all age 4+) on 22 November 2005. After fertilization, all eggs were pooled, incubated in EWOS hatching trays (45 × 45 × 20 cm) with flowing water (4.8 ± 0.3°C) and maintained in darkness. Two weeks before hatching, eggs were visually size sorted into two extreme size classes: “large” and “small” [10]. A total of 510 “small” eggs and 560 “large” eggs was selected for the experiment; the rest of the eggs were returned to the breeding program. Small and large eggs were incubated in three separate compartments (45 × 7 × 20 cm; [22]) with approximately 200 eggs in each compartment. At 95 days post fertilization, we measured egg diameter of a sample of 25 eggs per size class and a random sample of 50 eggs. This was done to validate our sorting into small and large egg size classes and compare those to the egg-size distribution in the population. The eggs were photographed and the diameter of each egg was later measured from these photographs using ImageJ software [53,54]. Egg sizes ranged from 31 to 50 mm representing egg sizes of Arctic charr from the Hólar breeding program ([53]; Fig 1). Small (S) eggs ranged from 30 to 37 mm (mean ± SD: 34.28 ± 1.70 mm) and differed from large (L) eggs ranging from 45 to 50 mm (mean ± SD: 47.16 ± 1.28 mm; t–test: t = 30.30, p-value < 0.0001). These selected eggs were representative of the lower and upper quarter of the size range (Fig 1). Eggs hatched over a period of eight days. The time to hatching, estimated as the time at which 50% of eggs had hatched [22], was 102 days after fertilization, and there was no significant difference in time of hatching between the egg size classes (t–test: df = 1; t = -1.35, p-value = 0.19). Light intensity was approximately 50 lux, and a 12:12 LD photoperiod was applied in order to mimick the natural light conditions in Iceland at this time of the year. Survival of fish in groups was above 90% from hatching until the end of the experiment. Among the individuals isolated since hatching, one S and one L fish died.

The time of first feeding, estimated as the time at which 50% of the fish had food in their digestive tracts (still visible at this stage of development), was 48 ± 7.1 days after hatching. There was no significant difference in time of first feeding between S and L fish (t–test: t = 1.05, p-value = 0.39), nor between fish raised at 4.5 °C or 6.5 °C (t–test: t = 1.02, p-value = 0.22). The age of the fish was counted from the time of hatching i.e. day post hatching (dph).

After the observations, all fish were returned to the ongrowing facilities.

### Temperature treatment

From fertilisation, all embryos were raised at 4.8 ± 0.3 °C. At 30 dph, half of the fish in each egg size class were assigned to 4.5 °C and the other half to 6.5 °C. Six individuals from each size class and from each temperature treatment were isolated and raised in similar rearing compartments as the fish maintained in groups ([22]; Table 1). Hereafter we refer to these individuals as the long-term isolated fish. Town tap water was used for this experiment. Other abiotic factors such as light intensity, photoperiod, water oxygenation (always > 100%) and water flow (0.2 cm.s^-1^) were kept constant for the two temperature treatments (see section above). In each hatching tray, water level was maintained at a height of 12 cm by a continuous freshwater supply.

**Table 1:** Design of the behavioural experiments performed on first-feeding Arctic charr (*Salvelinus alpinus*). Two experiments were performed. Fish were observed for foraging behaviour and mobility every week for four consecutive weeks (63, 70, 77, 84 days post hatching). Each weekly observation consisted of repeated measures of the individual over two days. Experiment A was conducted on fish raised in groups. Each observation was conducted on one fish (within a group of 6 fish) or one fish isolated 24 h before the observation (brief isolation). Naïve fish were used every week. Experiment B was conducted on fish isolated before hatching (i.e. long-term isolated, repeated measures on the same individuals).

**A-Individual observed in groups *versus* briefly isolated**			
	**Treatments**		**Replicates**
**Category of egg sizes**	**Temperature**	**Social environment**	**number of individuals observed each week**
small	4.5	group of 6 fish	6
small	4.5	brief isolation	6
large	4.5	group of 6 fish	6
large	4.5	brief isolation	6
small	6.5	group of 6 fish	6
small	6.5	brief isolation	6
large	6.5	group of 6 fish	6
large	6.5	brief isolation	6
**B-Long-term isolation of individuals (repeated behavioural observations of known individuals)**			
		**Treatments**	**Replicates**
**Category of egg sizes**	**Temperature**	**Social environment**	**number of individuals observed each week**
small	4.5	isolated since hatching	same 6 fish over time
large	4.5	isolated since hatching	same 6 fish over time
small	6.5	isolated since hatching	same 6 fish over time
large	6.5	isolated since hatching	same 6 fish over time

First-feeding fish were hand fed EWOS micro 013C (pellet size: 0.1 to 0.2 mm) twice a day, seven days per week, with a ration corresponding to 5% mean weight of juvenile Arctic charr. Food ratio was adjusted every week based on calculations of biomass in each compartment. Initial biomass was calculated based on the weight of 25 fish originating from small and large eggs raised at 4.5 and 6.5 °C in groups. Fish were individually anesthetized with 2-phenoxyethanol (3 ppm), measured to the nearest 0.1 mg and allowed to recover in aerated freshwater. This procedure was conducted every week after the observations started i.e. five times.

### Behavioural observations

To test for differences in foraging behaviour and mobility between fish from different treatments, we observed the fish in similar conditions as the rearing compartments (see above). Fish kept in isolation (i.e. long-term isolation) were also moved from their rearing compartments into similar compartments for observation to ensure similar handling of each individual. Fish were transferred to an observational tray and assigned to a social treatment one day before the trials (Table 1). Fish were not fed for 24 h to ensure a similar hunger level. Each fish was observed over two consecutive days for each week of observation. The experimental set-up was surrounded by opaque curtains in order to minimize fish disturbance and to allow the observer to be relatively hidden. Each trial consisted of a 30-second observation, followed by food distribution and a two-minute observation. Because the observer had a bird´s-eye view of the tank, we conducted direct observations to allow accurate localisation of the foraging behaviour [10]. Foraging behaviour was expressed as the number of foraging attempts [10]. Furthermore, we recorded the time when fish stayed immobile on the bottom. Immobility was expressed as the percentage of time spent immobile.

### Social treatment

We tested the effect of social environment by observing one fish maintained in a group of **six** fish *versus* fish put in isolation for the purpose of the observation (brief isolation; Table 1). After 24 hours, the observed individual in the group of six fish was chosen as the first fish crossing a randomly chosen area of the compartment a few seconds before the observation. Naive fish were used each week (age 63, 70, 77 and 84 dph) for fish in groups or briefly isolated. After each week, focal fish were raised in a different compartment and were never observed a second time. However, fish that were fully isolated (i.e. long-term isolated) were the same individuals. They were screened repeatedly each week for behaviour and this data set was analysed separately from the fish in groups or fish briefly isolated (see below).

### Data analyses

Statistical analyses were performed in R version 3.2.2 [55]. All tests were two–tailed with a significance level set to α = 0.05. As the behavioural data in this study exhibit an excess of zeros, we analyzed them using Hurdle models, which are designed to deal with the high occurrence of zeros in the data (see detailed description below).

#### Egg size and body size

Egg size (i.e. diameter) was normally distributed (Shapiro test: W = 0.97; p-value = 0.21) and showed homogeneity of variance (Levene test: F _1,48_ = 2,98; p-value = 0.09). Weight of fish in groups were log transformed and compared using an analysis of variance (ANOVA) with the full model: log (weight) ~ temperature × egg size × age. The selected order of the factors in this model was based on our prediction that temperature would have a larger impact on fish weight than egg-size categories and age (i.e. fish were measured every week). Hierarchical variance between the models was checked by running all possible order of factors of this 3-way ANOVA, which return essentially same results (see Results section). Growth of first-feeding fish was only assessed in fish kept in groups. We did not expect growth differences between fish isolated for the time of observations (isolated for only two days) and the fish raised in groups.

#### Behaviour variables

In this study, two types of behavioural data were analyzed using two-part models, one on fish foraging (bottom, water column, surface and total foraging) and another on fish activity (proportion of time spent immobile). All foraging variables were analysed separately, but “total foraging” represented the total number of all foraging events/attempts observed i.e. the sum of bottom, water column and surface foraging events. For each analysis, the explanatory variables included in the full model were social environment (group *vs*. briefly isolated), temperature (4.5 °C *vs*. 6.5 °C), age of fish (63, 70, 77 and 84 dph), egg-size group (small *vs*. large), and day of observation (two consecutive days; day1 *vs*. day2), as well as the interactions between social environment and temperature, social environment and egg-size group, and temperature and egg-size groups. The full model was reduced by backward selection based on the Akaike Information Criterion (AIC) [56]. Diagnostics based on residuals were performed to assess the adequacy of the reduced model and compliance to the underlying assumptions. Dependent variables were transformed whenever necessary to ensure that the residuals followed the assumed error distribution. Finally, the effects of the independent variables were estimated from the reduced models and their significance was tested by likelihood ratio tests (LRT) between nested models respecting marginality of the effects that are supposed to follow a *χ*^2^ distribution under the null hypothesis (type II tests; [57]).

All foraging behaviour variables (bottom, water column, surface and total foraging) were analyzed using Hurdle Negative Binomial models (HNB) as the data collected here were count data with excess zeros. These models are called two-part models: the first part is a binomial probability model that governs the binary outcome of whether a count variable has a zero or a positive value and the second part is a zero-truncated count model that governs the positive outcomes [58]. In this study, this type of model allowed u**s** to test the effects of the independent variables cited above on (i) the probability of fish that forage/move *versus* the ones that do not, and (ii) to test the effects of the same factors only among the fish that were mobile or foraged.

Fish activity was analysed using two-part GLM models. The first part is a binomial probability model that governs the binary outcome of whether the activity of fish has a zero or a positive value (i.e. mobile or immobile), and the second part is a normal distribution on the positive outcomes (only considering the fish that showed immobility). Percentage of time spent immobile (i.e. fish activity) was log transformed. The independent variables in the full model were similar to the one for the foraging variables.

As described above, fish observed in groups or briefly isolated were analysed together i.e. naive fish were used at each age (Table 1). Observations of long-term isolated fish were repeated data on similar individuals over time and therefore were analyzed separately from the two other social categories (Table 1). These observations were analyzed using generalized linear mixed-effects models (GLMM). A random effect that affected the model intercept only was used to account for variability due to individuals. In this case, the full model was reduced by backward selection in two steps: the random part of the model was reduced first and the fixed part was selected afterwards [56,59] Selection was based on significance of the effects at a 5% alpha risk threshold determined by likelihood ratio tests (LRT) between nested models while respecting the marginality of the effects. The GLMM models were fitted using the package *lme4* in R [60].

The full model included individual as a random effect and rearing temperature (4.5 °C *vs*. 6.5 °C), age of fish (63, 70, 77 and 84 dph), egg size group (small *vs*. large), and day of observation (two consecutive days; day1 *vs*. day2), as well as the interaction between temperature and egg-size group as fixed effects.

No significant variability due to individuals, regardless of variable studied, was observed (S1 and S2 Tables). Therefore, random effect was removed from the full model. HNB models for the foraging variables and two-part GLM models for fish activity were used as described above. In most graphs dotted lines were fitted to depict the 50% probability of feeding or immobile fish in order to convey a biological meaning to the data. The two-part models, HNBs and GLMs, were fitted using the *pscl* and *stats* packages [55,61,62].

## Results

### Growth

**Fig 2:**
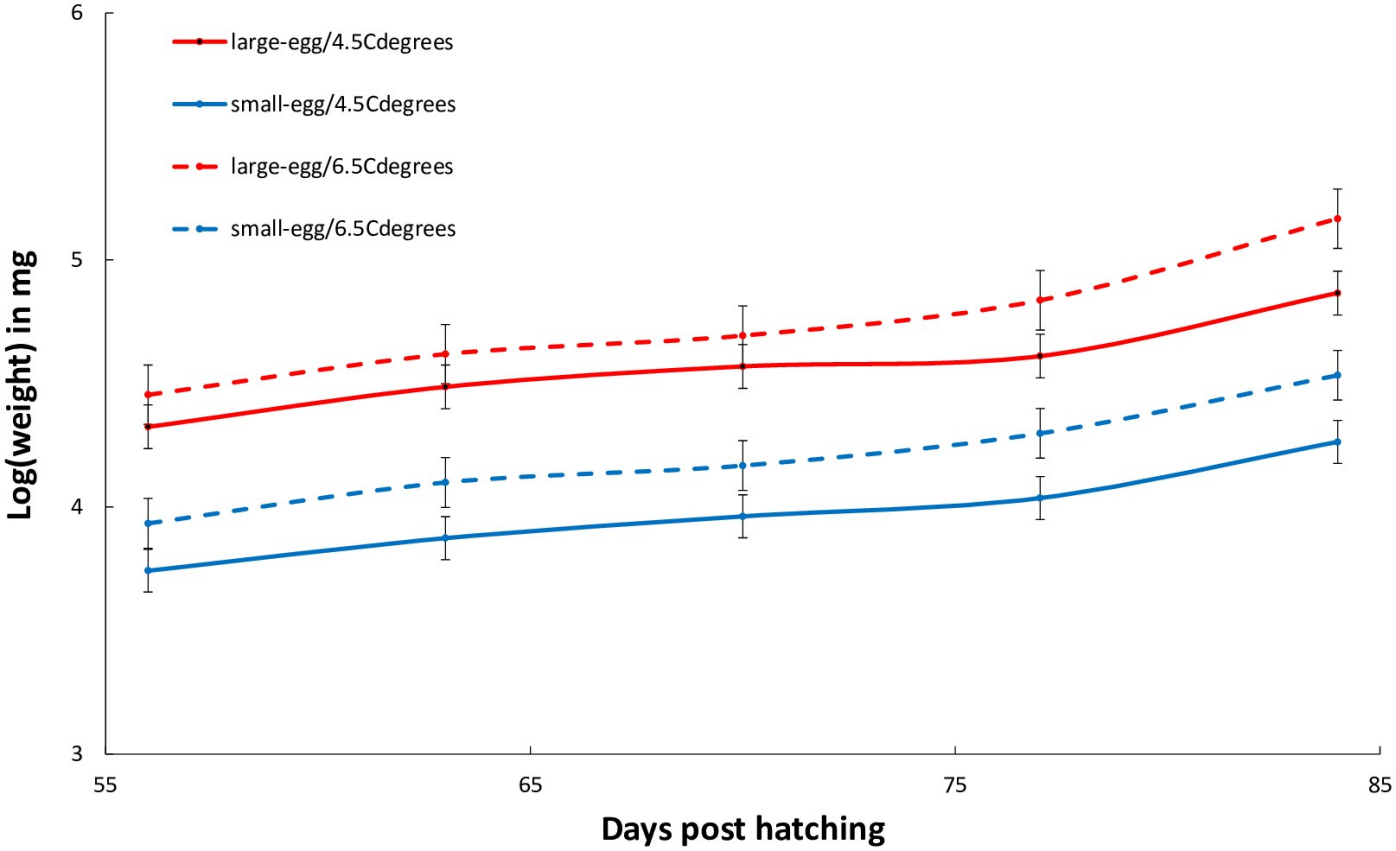
Growth trajectories of first-feeding Arctic charr (*Salvelinus alpinus*; fish coming from small and large eggs) reared at 4.5 and 6.5 °C. Means and standard deviations of the mean (SD) are shown (n = 500). The lines connect the means. Temperature treatment was started 30 days post hatching (dph) and time to first feeding for all groups was 48 dph.

Body weight of first-feeding fish increased with age in all treatments (Fig 2; Table 2). At the start of the experiment, fish hatching from large eggs were larger than fish hatching from small eggs within each temperature treatment (Table 2; Fig 2). A gradient was observed as large fish from large eggs at 6.5 °C were larger than those at 4.5 °C, themselves being larger than small fish coming from small eggs raised at 6.5 °C, those being larger than small fish coming from small eggs raised at 4.5°C. When looking at each of the five time points, all pairwise comparisons of the interaction between temperature and size were significant (all pairwise comparisons with p-value ≤ 0.05; Table 2; Fig 2). A significant three-way interaction between temperature, egg size and age was seen because some fish did not show significant growth between 63 and 70 dph (e.g. small fish raised at 6.5 °C and 4.5 °C). Growth trajectories of fish originating from large and small eggs were very similar within each temperature. There was no indication that smaller fish raised at higher temperature showed higher growth than larger fish raised at lower temperature (Fig 2).

**Table 2:** Results of the ANOVA model for growth of juvenile Arctic charr raised in a benign environment at 4.5 and 6.5 °C, originating from two egg-size classes. Body weight (mg) data were log transformed to meet the assumptions of normality and homogeneity of variance (using Shapiro and Levene tests respectively; adjusted R^2^ = 0.91). Egg size refers to the egg-size class the fish originated from: “small” or “large” eggs. Fish were raised at 4.5 or 6.5 °C degrees and measured for body weight five times during development, at 57, 63, 70, 77 and 84 days post hatching. (n = 500).

	d.f.	F value	*P*	
Temperature	1	492.35	< 0.0001	***
Egg size	1	3457.43	< 0.0001	***
Age	1	1656.28	< 0.0001	***
Temperature × egg size	1	5.28	< 0.0500	*
Temperature × age	1	28.38	< 0.0001	***
Egg size × age	1	1.43	0.2339	
Temperature × egg size × age	1	5.54	0.0500	

p-values 0.01 ≤*≤ 0.05, and p-values 0.001 ≤ are indicated with ***

### Mobility and foraging behaviour for fish raised in groups vs briefly isolated

**Fig 3:**
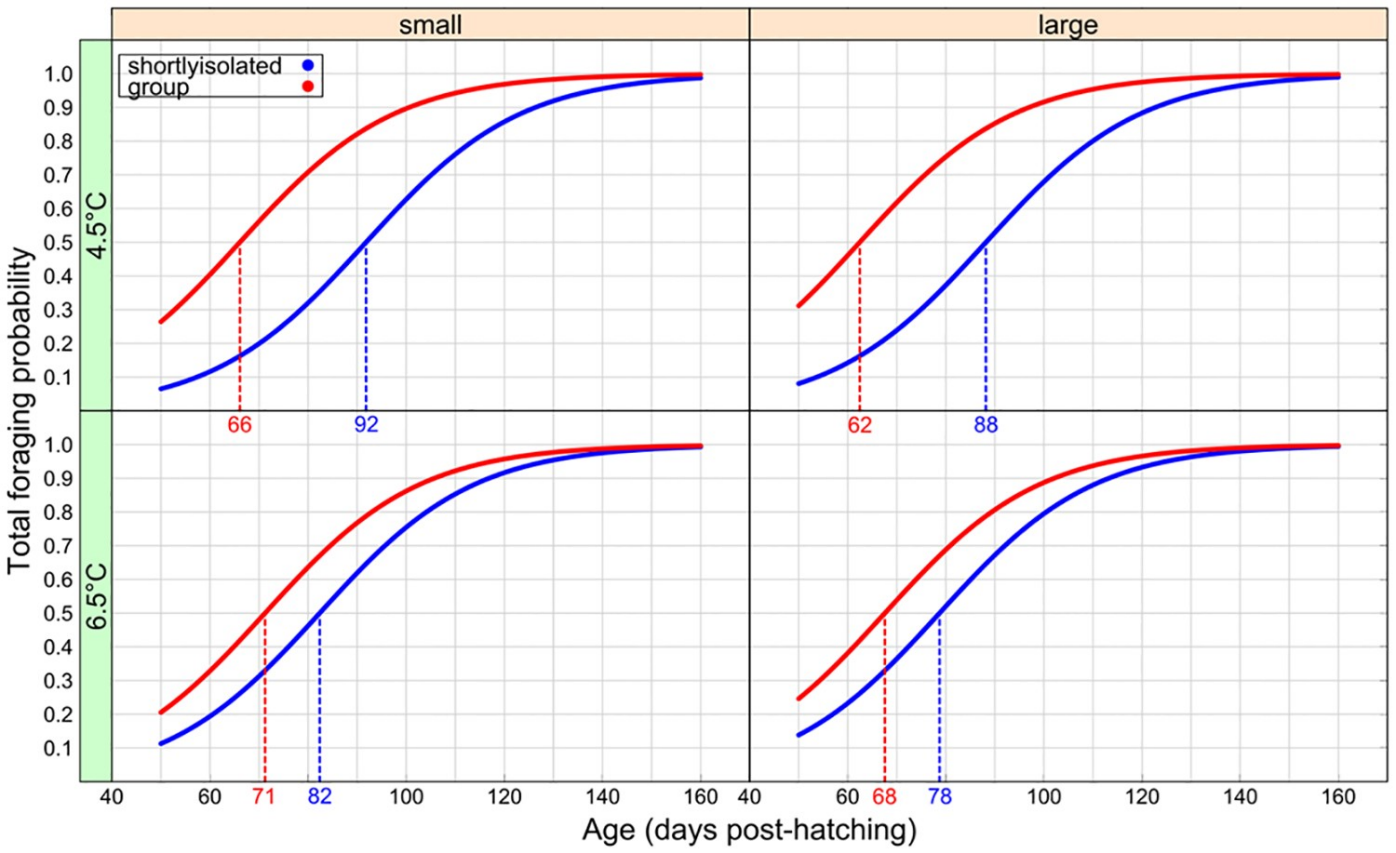
Effect of temperature, egg size and social environment on foraging probabilities of first-feeding Arctic charr (*Salvelinus alpinus*). Curves represent the estimated probability of foraging fish per social rearing environment (group in blue and briefly isolated in red), and rearing temperature (in rows) as function of age (days post hatching). The dotted lines indicate the projected age at which 50% of fish are feeding. The color of the dotted lines color refers to the social environment.

The probability of foraging (“Total foraging”) was mainly determined by an interaction between temperature and social environment (Social × Temperature effect, Table 3; Fig 3). At any given age, the probability of foraging fish was higher in groups than in fish placed in brief isolation at both 4.5 °C and 6.5 °C (Social effect, Table 3; Fig 3). However, the difference of probability of foraging fish between social environments was significant at 4.5 °C (post hoc test: z-value = -4.877, p-value < 0.001, Fig 3) and was not significant at 6.5 °C (post hoc test: z-value = -2.314, p-value = 0.071, Fig 3). At 4.5 °C, a difference in feeding date (50% of fish feeding) of 26 days was observed between social treatments, for both small and large fish (Fig 3). At 6.5 °C this difference was only 11 and 10 days respectively (Fig 3) and was the consequence of an increase of the foraging probability in fish briefly isolated while it remained relatively constant in fish raised in groups. The probability of foraging also increased with age but did not vary with egg size (Table 3). Finally, fish in groups were more likely to feed than fish that were briefly isolated. This was seen at all levels of the water column (Social effect, Table 2; Fig 3). Considering where in the water column the foraging took place, egg size, age and social environment significantly affected the probability of foraging fish. For all locations in the water column, foraging probability increased with age (Table 3). Also, the larger the egg (i.e. the longer and heavier the fish [10]) the higher the probability of foraging in the water column and at the surface (Table 3).

**Table 3:** Parameter estimates for best-supported Hurdle Negative Binomial (HNB) models predicting the count and the probability of foraging behaviours of newly feeding Arctic charr (*Salvelinus alpinus*). Both outcomes of the models are reported: the first part (“Binomial error distribution”) is a binomial probability model that governs the binary outcome of whether a count variable has a zero (no foraging) or a positive value (foraging) and the second part (“Negative binomial error distribution) is a zero-truncated count model that governs the positive outcomes (tested the importance of the independent variable only among the feeding fish”). The variable “Total foraging” is the sum of all foraging attempts at all locations in the water column: bottom foraging, water column and surface foraging.

*Variable*	*Effect*	*Binomial error distribution (logit link)*					*Negative binomial error distribution (log link)*				
		*Estimate*	*s*.*e*.	*df*	*χ*^2^	*p-value*	*Estimate*	*s*.*e*.	*df*	*χ*^2^	*p-value*
Bottom foraging	Intercept (large/group)	-2.552	1.094	n.a	n.a.	n.a.	-1.824	1.053	n.a	n.a.	n.a.
	Age	0.030	0.015	1	4.288	**0.038**	0.030	0.013	1	4.341	**0.037**
	Size (small)	0.131	0.300	1	0.727	0.394	0.438	0.222	1	2.005	0.157
	Social (briefly isolated)	-0.410	0.307	1	11.288	**<0.001**	0.244	0.251	1	0.030	0.862
	Social × Size	-0.746	0.459	1	2.669	0.102	-0.507	0.373	1	1.822	0.177
Water column foraging	Intercept (4.5C/large/day1/group)	-11.011	1.641	n.a	n.a.	n.a.	-4.273	2.73	n.a	n.a.	n.a.
	Age	0.131	0.021	1	49.058	**<0.001**	0.057	0.032	1	4.103	**0.043**
	Temperature (6.5C)	0.642	0.288	1	5.097	**0.024**	0.331	0.349	1	0.966	0.326
	Size (small)	-0.903	0.292	1	10.04	**0.002**	-0.257	0.334	1	0.671	0.413
	Date (day2)	0.633	0.287	1	4.967	**0.026**	-0.411	0.315	1	1.754	0.185
	Social (briefly isolated)	-0.946	0.292	1	11.043	**<0.001**	-0.447	0.365	1	0.69	0.406
Surface foraging	Intercept(4.5C/large/group)	-4.78	1.858	n.a	n.a.	n.a.	-5.50	3.203	n.a	n.a.	n.a.
	Age	0.045	0.024	1	3.543	0.060*	0.071	0.040	1	3.336	0.068*
	Size (small)	-1.119	0.410	1	8.207	**0.004**	0.250	0.542	1	0.060	0.806
	Social (briefly isolated)	-1.127	0.410	1	8.581	**0.003**	-1.12	0.818	1	2.566	0.109
Total foraging	Intercept (4.5C/day1/group)	-4.394	1.118	n.a	n.a.	n.a.	-1.877	0.764	n.a	n.a.	n.a.
	Age	0.065	0.015	1	19.75	**<0.001**	0.037	0.010	1	16.221	**<0.001**
	Temperature (6.5C)	-0.323	0.313	1	0.281	0.596	0.303	0.150	1	7.485	**0.006**
	Date (day2)	0.477	0.228	1	4.437	**0.035**	-0.163	0.124	1	1.585	0.208
	Social (briefly isolated)	-1.652	0.339	1	26.957	**<0.001**	-0.287	0.236	1	1.056	0.304
	Social × Temperature	0.937	0.457	1	4.253	**0.039**	0.214	0.286	1	0.564	0.453

p-values < 0.05 are in bold, p-values 0.05 <*<0.1 are nearly significant.; n.a. indicates that this particular factor(s) were not retained after model selection.

The number of foraging fish increased with age for all foraging variables (number of total foraging attempts and the number of foraging attempts at three locations; Table 2; negative binomial distribution). Out of all explanatory variables, only temperature significantly affected the total number of foraging events/attempts, which was higher at 6.5 °C (Table 3).

**Fig 4:**
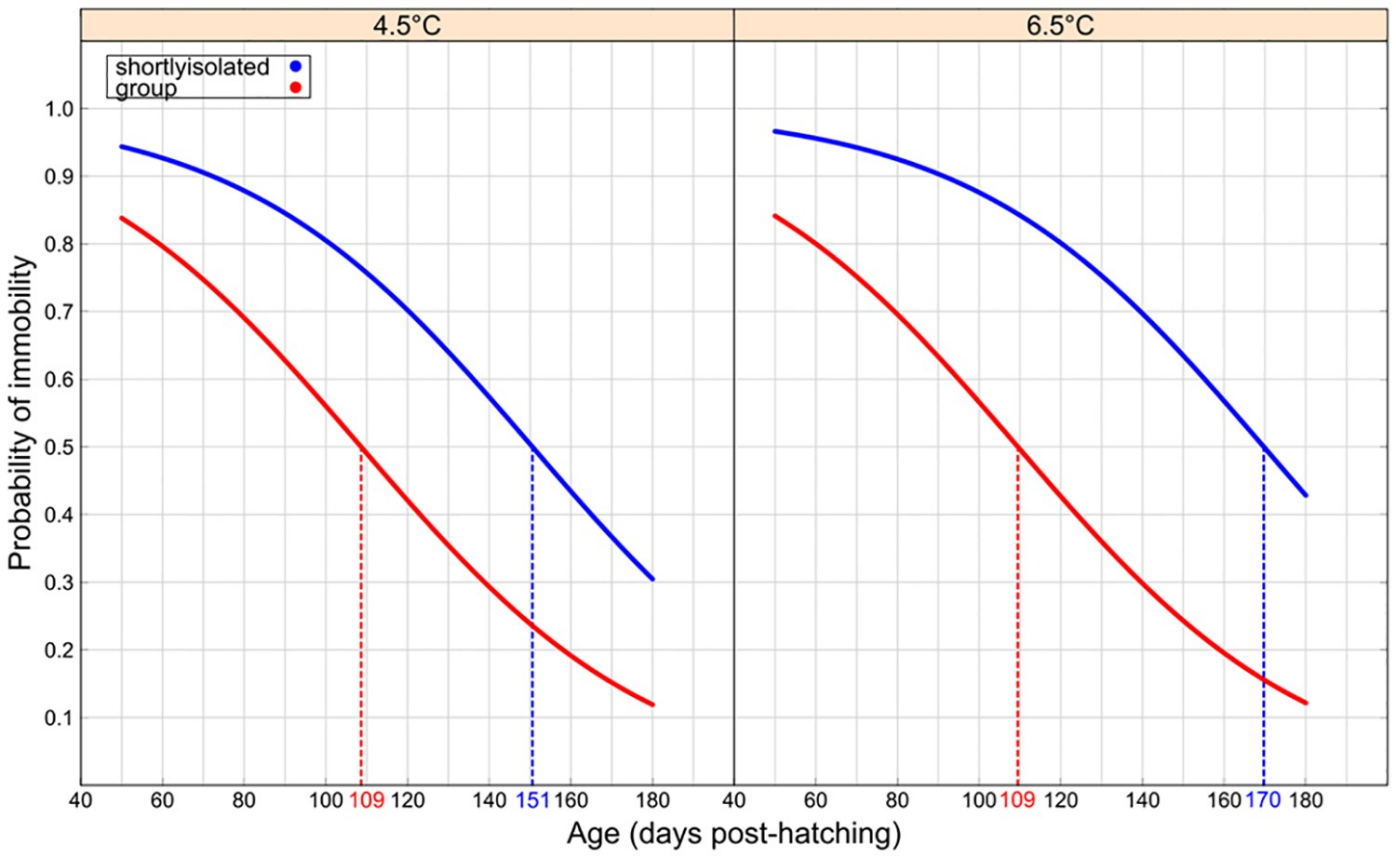
Effect of temperature, egg size and social environment on immobility probabilities of first-feeding Arctic charr (*Salvelinus alpinus*). Curves represent the estimated probability of immobility per social rearing environment (group in blue and briefly isolated in red) as function of age (dph). The dotted lines indicate the projected age at which 50% of fish are mobile. The color of the dotted lines refers to the social environment.

During the first week of observation, more than 75% of the fish were immobile regardless of temperature, egg size and social environment. At all times, fish raised in groups were more mobile than fish isolated for the observations (Table 4; Fig 4). The threshold of 50% mobile fish was reached at age 109 dph in fish raised in groups against 160 dph in fish raised in brief isolation (Fig 4). Fish raised at a colder temperature and isolated were more immobile than fish raised at a higher temperature and in groups (post hoc test 4.5 °C/ briefly isolated vs. 6.5 °C/group: z-value = 4.679, p-value ≤0.001, Fig 4). Size did not influence mobility of newly feeding Arctic charr.

**Table 4:** Parameter estimates for best-supported two-part GLM models predicting the percentage and the probability of immobility in newly feeding Arctic charr (*Salvelinus alpinus*).

*Effect*	*Binomial error distribution (logit link)*					*Normal error distribution (identity link)*				
	*Estimate*	*s*.*e*.	*df*	*χ*^2^	*p-value*	*Estimate*	*s*.*e*.	*df*	*χ*^2^	*p-value*
Intercept (4.5C/large/group)	3.376	1.392	n.a.	n.a.	n.a.	5.693	0.563	n.a.	n.a.	n.a.
Age	-0.028	0.018	1	2.372	0.124	-0.026	0.008	1	11.916	**<0.001**
Temperature (6.5C)	n.a.	n.a.	n.a.	n.a.	n.a.	-0.454	0.177	1	3.742	0.053*
Size (small)	-0.592	0.338	1	1.298	0.2545	n.a.	n.a.	n.a.	n.a.	n.a.
Social (briefly isolated)	0.934	0.433	1	22.944	**<0.001**	0.344	0.172	1	21.423	**<0.001**
Social × Temperature	n.a.	n.a.	n.a.	n.a.	n.a.	0.406	0.239	1	2.884	0.089*
Social × Size	0.969	0.646	1	2.292	0.130	n.a.	n.a.	n.a.	n.a.	n.a.

p-values < 0.05 are in bold; p-values 0.05 <*<0.1 are nearly significant; n.a. indicate that this particular factor was not retained after model selection.

### Mobility and foraging behaviour of individuals raised in long-term isolation

The same individuals were tested over time for mobility and feeding behaviour, and this was accounted for using the individual’s ID as a random factor in the HNB models (see Methods). However the random factor “individual” was never significant, indicating that there was no consistency in behaviour within individuals over time.

**Fig 5:**
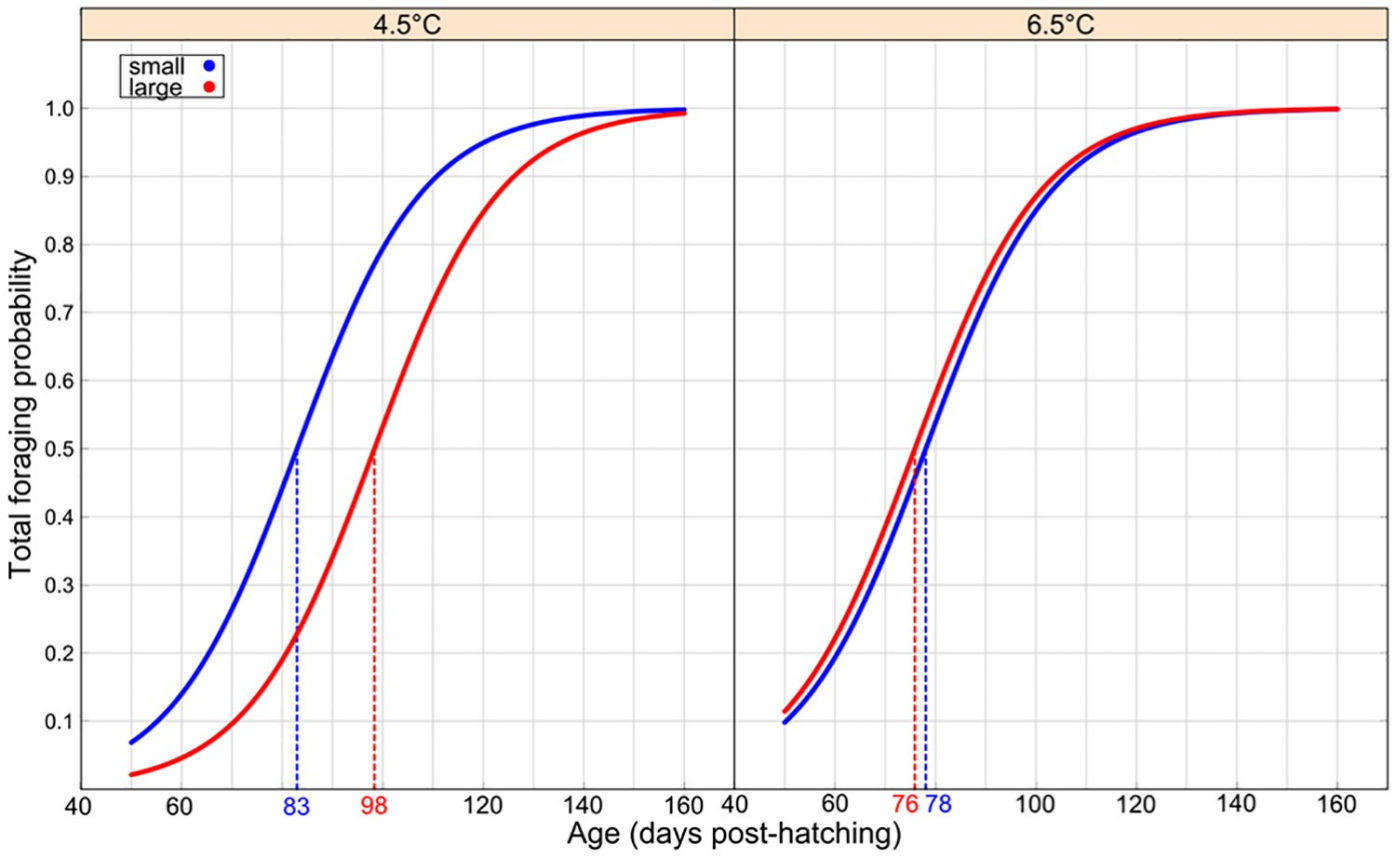
Effect of temperature and egg size on foraging probabilities of first-feeding Arctic charr (*Salvelinus alpinus*) raised in long-term isolation. Curves represent the estimated probability of total foraging attempts per fish according to egg size/body size (small and large individuals), and rearing temperature (columns) as a function of age (days post hatching). The dotted lines indicate the projected age at which 50% of fish are feeding. The color of colored lines refers to initial egg-size categories.

Overall, surface foraging was observed only four times and only at 6.5 °C; therefore, the HNB model did not converge for this foraging variable. The probability of foraging, regardless of the location, increased with age (Table 5, left column). The probability of foraging on the bottom and the probability of foraging attempts (“Total foraging”) were predominantly determined by an interaction between temperature and size (Temperature × Size effect, Table 5). At 4.5 °C, the model predicted that 50% of small fish were feeding at 83 dph whereas fish coming from larger eggs started feeding at approximately 98 dph (Fig 5), but such differences were not seen at 6.5 °C (Table 5; Fig 5). Large fish raised at 6.5 °C started feeding 22 days earlier than those raised at 4.5 °C (post hoc test: z-value = 2.999 and p-value = 0.014, Fig 5). Small fish raised at 6.5 °C fed earlier than larger ones at 4.5 °C (respectively 78 and 98 dph, post hoc test: z-value = 2.701 and p-value = 0.034, Fig 5). Finally, none of the factors of interest affected the foraging behaviour of individuals among the feeding fish raised in isolation (right column of Table 5).

**Table 5:** Parameter estimates for best-supported Hurdle Negative Binomial (HNB) models predicting the probability of foraging behaviours of newly feeding Arctic charr (*Salvelinus alpinus*). All fish were kept in isolation from hatching. Both outcomes of the models are reported: the first part (“Binomial error distribution”) is a binomial probability model that governs the binary outcome of whether a count variable has a zero (no foraging) or a positive value (foraging) and the second part (“Negative binomial error distribution”) is a zero-truncated count model that governs the positive outcomes (tested the importance of the independent variable only among the feeding fish). The variable “Total foraging” is the sum of all foraging attempts at all locations in the water column: bottom foraging, water column and surface foraging (see note).

*Variable*	*Effect*	*Binomial error distribution (logit link)*					*Negative binomial error distribution (log link)*				
		*Estimate*	*s*.*e*.	*df*	*χ*^2^	*p-value*	*Estimate*	*s*.*e*.	*df*	*χ*^2^	*p-value*
Bottom foraging	Intercept (4.5C/ large)	-5.806	1.840	n.a.	n.a.	n.a.	0.390	1.799	n.a.	n.a.	n.a.
	Age	0.053	0.024	1	5.133	**0.023**	0.001	0.024	1	0.018	0.893
	Temperature (6.5C)	1.390	0.586	1	2.466	0.116	0.297	0.538	1	0.281	0.596
	Size (small)	1.079	0.590	1	0.440	0.507	0.423	0.549	1	0.038	0.846
	Temperature × size	-1.453	0.764	1	3.783	0.052*	-0.748	0.676	1	1.142	0.285
Water column foraging	Intercept (4.5C/day1)	-14.070	3.529	n.a.	n.a.	n.a.	1.243	4.227	n.a.	n.a.	n.a.
	Age	0.147	0.044	1	15.155	**<0.001**	-0.015	0.050	1	0.088	0.766
	Temperature (6.5C)	1.055	0.585	1	3.519	0.061*	0.894	0.784	1	2.457	0.117
	Date (day2)	0.137	0.549	1	0.062	0.803	-1.354	0.620	1	6.056	**0.014**
Total foraging	Intercept (4.5C/ large)	-7.775	1.872	n.a.	n.a.	n.a.	-0.002	1.736	n.a.	n.a.	n.a.
	Age	0.079	0.024	1	11.204	**<0.001**	0.005	0.023	1	0.166	0.683
	Temperature (6.5C)	1.776	0.592	1	7.414	**0.006**	0.466	0.569	1	0.123	0.726
	Size (small)	1.217	0.596	1	1.206	0.272	0.544	0.589	1	0.047	0.829
	Temperature × Size	-1.389	0.761	1	3.490	0.062*	-0.833	0.686	1	1.482	0.224

p-values < 0.05 are in bold, p-values 0.05 <*<0.1 are nearly significant.

Model did not converge for the variable surface foraging, therefore this variable is not included in the table.

The probability of being immobile tended to be lower in large individuals (df = 1, *χ*^2^ = 2.775, p-value = 0.096), and mobility increased with temperature (df = 1, *χ*^2^ = 17.586, p-value< 0.001).

## Discussion

The aim of this study was to assess the interactive effect of egg size, social condition and temperature on early body size and behaviour of Arctic charr *S*. *alpinus*, a cold-water species, at early life stages of development. As predicted, the observed growth resulted from the interaction of temperature and egg size of origin, but we found that small fish raised at a higher temperature did not grow faster than large fish raised at a lower temperature. Behavioural responses were also driven by interactive factors, i.e. temperature affected the behaviour of fish differently according to body size and/or social context. Below, we further discuss the implications of our findings in the context of subtle temperature differences and interpret our results in a context of rapid changes in temperature (in connection to e.g. anthropogenic changes seasonal variation) with an emphasis on the potential consequences for the evolution and ecology of highly diverse northern freshwater species.

### Growth trajectories and behaviour of fish raised in groups or briefly isolated

Overall, whatever the conditions and at all ages, fish from large eggs were larger than those from small eggs, and that remained true from the start of the experiment. This size difference in Arctic charr raised in benign conditions provided by the hatchery environment may persist over a very long period of time unlike other salmonids [15] in which body size differences tend to be transient and limited to the initial period following hatching (reviewed by [8], but see [63]). The effects of egg size for individual phenotypes may strongly depend on environmental factors. In our experiment we expected temperature, a crucial determinant of foraging and growth in all ectothermic animals through its effects on metabolism [33], to interact with egg size in determining Arctic charr growth. First of all, contrary to our predictions, small fish raised at a higher temperature did not grow faster than large fish raised at a lower temperature. In fact, growth trajectories of fish raised at 4.5 and 6.5 °C were very similar up to the end of the experiment (Fig 2). The observed growth trajectories between size classes are likely to persist in time regardless of the temperature treatment. Therefore, this study revealed that maternal effect (i.e. egg size) impacted growth of first-feeding fish, and these effects were not cancelled out by a small but constant warmer temperature. These results add to the extensive literature on the importance of the interaction of temperature and body size for growth of fish (e.g. [10,64]). Importantly, the temperature treatment applied here was subtle (increase of 2 °C applied over a few weeks at first feeding) and therefore differs with most literature on cold-water fishes. Our data emphasise the need for future research specifically looking at growth of first-feeding fish in the context of small increases in temperature and associated metabolic rate.

Behavioural responses were also driven by interactive factors that differed according to the variables. Overall, the probability of foraging and mobility were higher for fish in groups than in brief isolation. This was observed at both temperatures, which confirms the importance of social environment for behavioural ontogeny in juvenile Arctic charr [10]. The probability of surface foraging was higher in large fish and in fish raised in a group compared to those briefly isolated (see also [10,22]). Fish were overall more mobile and foraged more at 6.5 than at 4.5 °C. A temperature increase induces higher swimming activity in fish when it is in the preference range of the species, as it has been shown in lab conditions (e.g. [65]) or in the wild (e.g. [66]). The increase in foraging probability could be explained by an increase in metabolism and energy demand [34,35]. Previous studies have shown an increase in the number of foraging events [36,67–70], the amount of time spent feeding [34,36,67–70] and the distances covered during feeding [35,71] at higher temperature. However, to the best of our knowledge, the present study is the first to show an impact of a small (2 °C) increase on behaviour of Arctic charr juveniles, adding to the evidence that even small, rapid increases or fluctuations in temperature within the thermal tolerance of a species can affect behaviour of fishes (e.g. [35] during ontogeny).

Interestingly, the foraging probability difference between fish raised in groups and fish briefly isolated was higher at 4.5 °C than at 6.5 °C for both small and large fish. Previous studies have shown that fish become more active and aggressive at higher temperatures [34,35], or tend to spend more time schooling and inspecting in the presence of a predator [72,73], but very few have evaluated the importance of both social environment and temperature increase. Our results indicate that there is no additive effect of temperature and social environment shortly after feeding. This can be explained by social interactions that may limit foraging activities in a group of fish, whereas the isolated fish increase their feeding with temperature without limitations by social cues. Fish in isolation are not limited by the presence of congeners or food shortage, and therefore increase their probability of foraging as a response to higher metabolism driven by the temperature increase [34,35]. Importantly, no aggressive behaviour was reported in this study at any temperature. As shown in previous studies, metabolic rate scales allometrically with both temperature and body size in fish [74,75], which is true to a larger extent for all vertebrates [76]. In our study, changes in behaviour in response to a subtle temperature change might be due to the relatively larger change in metabolic rate experienced by large fish i.e., a 2 °C temperature change will lead to different magnitudes of change in the metabolism of a small vs. large fish. This might be the driver of the increased foraging observed in large fish. Further studies linking behaviour and metabolism are required to confirm these hypotheses.

### Behaviour of long term isolated fish

Fish isolated since hatching displayed different behaviour than those that were briefly isolated, although the comparison was not specifically addressed in this study (Table 1). In full isolation, fish were rarely observed foraging at the surface, and only at 6.5 °C. A previous study showed that fish isolated since hatching were less mobile and exhibited lower foraging activity than fish held in brief isolation or in a group [10], which can be explained by the absence of social stimulation [77]. The probability of foraging increased with age, which is in line with the higher amount of food needed to fulfill metabolic requirements [10]. Overall, foraging probabilities were explained by an interaction of temperature and size. Small fish raised at at 4.5 °C started to feed earlier than larger fish, which was not the case at 6.5 °C. At 6.5 °C, large fish started to feed 22 days earlier than their conspecifics at lower temperature, but small fish only slightly modified their probability of foraging between the two temperatures. This is likely associated with lower mobility observed in smaller fish. Smaller fish might cope with the temperature increase without modifying their energy demand because of their reduced activity in comparison to larger fish. This could be related to a sit-and-wait strategy as observed in many salmonids species wherein individuals wait to ambush approaching prey [78]. On the contrary, the foraging behaviour of the large fish observed in our study could be related to a different strategy of foraging in the wild wherein individuals actively seek prey. Previous studies have shown a positive relationship between energy demand and activity in juvenile Atlantic salmon (*Salmo salar*) [79]. Overall mobility increased with temperature in both small and large fish but was higher in large ones. Finally, we can raise the same mechanistic hypothesis as described in the previous section. Metabolic rate scales allometrically with both temperature and body size in fish so that the larger change in metabolic rate experienced by larger fish could explain differences in growth (although not tested here) and foraging behaviour including date of first feeding. Interestingly, we also detected no consistency in foraging and mobility behaviour within individuals, indicating that the behaviour of first feeding Arctic charr is highly variable among and within individuals.

### Early phenotypic differences in the context of global warming

The fact that temperature did not affect the foraging behaviour of small and large fish in the same way is important. Temperature in ectothermic animals has been thought to be the ultimate governing factor for survival, development (including growth), metabolism and activity of fishes [24]. This may explain why so little research has been done on temperature effect in interaction with other factors, especially at early developmental stages, and how those can have a potential impact on other phenotypic traits than growth. Here we show that a difference of 2 °C applied over less than two months resulted in much earlier foraging activities in larger fish only (Fig 5). This may be linked to differences in metabolic rate which can vary greatly among individuals [80]. These findings can have implications in terms of behaviour (and growth) of first-feeding salmonids, especially in shallow lakes where a 2 °C difference in water temperature may represent an increase in temperature over just a few days or weeks. Larger fish may be more plastic than smaller ones in response to a small increase of temperature as seen here in behaviour and growth (also seen in brook trout *Salvelinus fontinalis* [81]).

In the wild, differences in plasticity based on individual size may result in different behavioural strategies in foraging behaviour and mobility of individuals within a population. In polymorphic salmonids, morphs are distinguished by resource use and associated foraging behaviour, as well as the decision to disperse or migrate from the spawning grounds. In this context, our findings may indicate that individuals that differ in size may respond differently to a subtle temperature increase during crucial ontogenic events such as first feeding in fishes.

In this study we tested the effect of a subtle but realistic difference of 2 °C in temperature on behaviour and growth of fish. Such temperature variation may be experienced on a daily basis by young salmonids found in lakes and streams, with variation in water temperature between day and night or between pools and riffles, or in the context of global warming. Few studies have estimated how subtle differences of temperature may affect organisms at several phenotypic levels and how this factor can interact with other important factors at early developmental stages. Here we demonstrated that a subtle increase in temperature did not affect behaviour and growth of individuals in the same way depending on their size. Even more importantly, when significant effects of temperature and initial egg size were detected on growth of young fish, there was no indication of compensatory growth in smaller fish raised at high temperature in comparison to larger ones raised at 4.5 °C. This is rather surprising as small fish from small eggs tend to grow faster than their counterparts after hatching [63,82]. Smaller fish may emerge earlier from the gravel because they have a smaller yolk sac [53] and often develop faster [82]. Higher growth rate in smaller fish, leading sometimes to compensatory growth, has been linked to higher expression of growth hormone receptors in cichlids [83]. For Arctic charr, a northern freshwater species, subtle difference in temperature, social context and egg size resulted in different phenotypes of individuals in a controlled environment. Clearly, our results call for quantifying how metabolism scales with a small increase in temperature in Arctic charr along with behaviour and growth.

The changes in individual phenotypes shown in this study may have implications for better understanding what shapes intraspecific diversity of aquatic species, as well as for the management and conservation of those species. The relevance of these results would need to be tested in combining field and lab experiments, although controlling for stability of temperature during early development of salmonids may be a very tedious task in animals that take several months to develop and start feeding. Further studies linking behaviour and metabolism at the individual level are required to better understand the ecological and evolutionary importance of individual variation observed in our study.

The behavioural and growth changes induced by the 2 °C difference can be interpreted in the context of global warming. The increasing temperature of habitats occupied by Arctic charr, combined with initial size differences between individuals, could modify behaviour and growth trajectories of this northern freshwater species. Modification of mobility and foraging probabilities of individuals will particularly affect life history traits and resource use (habitat and food). In a species like Arctic charr in which numerous examples of resource polymorphism have been shown, one can expect that a subtle difference in temperature (even if comprised within the temperature tolerance of the species) combined with other factors will affect the evolution of individual phenotype resulting in modification of observed polymorphism. Environmental changes have been seen to directly affect polymorphism such as collapses of sympatric morphs [84], but surprisingly none have yet been linked to temperature. To better predict the magnitude of the effects connected to a temperature increase for phenotypes of northern freshwater fishes, more research combining ecology, evolution and development of individuals is needed, especially direct observation in the field. Such study would yield valuable results in the field of ecology, evolution and conservation of northern freshwater species.

## Supporting information

S1 Dataset Data set for behavioural variables. (TXT)

S2 Dataset Data set for growth. (TXT)

S1 Table Random effects testing using Likelihood Ratio Test (LRT). The analysed variables are the foraging behaviours (bottom foraging, water column foraging, surface foraging and total foraging) of long-term isolated fish.
(DOCX)

S2 Table Random effects testing using Likelihood Ratio Test (LRT). The analysed variable is the immobility of long-term isolated fish.
(DOCX)
