# Supplementary material for: Subtle temperature increase can interact with individual size and social context in shaping phenotypic traits of a coldwater fish
Source: PLoS One. 2019 Mar 27;14(3):e0213061. doi: 10.1371/journal.pone.0213061 (PMC6436715; doi:10.1371/journal.pone.0213061)
Supplement: S1 Table — The analysed variables are the foraging behaviours (bottom foraging, water column foraging, surface foraging and total foraging) of long-term isolated fish. (DOCX) [file pone.0213061.s003.docx]

**S1** **Table.** Random effects testing using Likelihood Ratio Test (LRT). The analysed variables are the foraging behaviours (bottom foraging, water column foraging, surface foraging and total foraging) of long-term isolated fish.

| ***Variable*** | ***Random effects*** | ***Random effects sources*** | ***Binomial error distribution (logit link)*** | | | ***Negative binomial error distribution (log link)*** | | |
| --- | --- | --- | --- | --- | --- | --- | --- | --- |
|  |  |  | ***df*** | ***$\chi2$*** | ***p-value*** | ***df*** | ***$\chi2$*** | ***p-value*** |
| Bottom foraging | Intercept | Individual | 1 | 1.276 | 0.2586 | 1 | 0.148 | 0.700 |
| Water column foraging | Intercept | Individual | 1 | 0 | 1 | 1 | 0 | 1 |
| Total foraging | Intercept | Individual | 1 | 1.437 | 0.231 | 1 | 0.594 | 0.441 |

p-values < 0.05 are in bold.

Model did not converge for the variable surface foraging, therefore this variable is not included in the table.
