# Supplementary material for: Subtle temperature increase can interact with individual size and social context in shaping phenotypic traits of a coldwater fish
Source: PLoS One. 2019 Mar 27;14(3):e0213061. doi: 10.1371/journal.pone.0213061 (PMC6436715; doi:10.1371/journal.pone.0213061)
Supplement: S2 Table — The analysed variable is the immobility of long-term isolated fish. (DOCX) [file pone.0213061.s004.docx]

**S2 Table.** Random effects testing using Likelihood Ratio Test (LRT). The analysed variable is the immobility of long-term isolated fish.

| ***Variable*** | ***Random effects*** | ***Random effects sources*** | ***Binomial error distribution (logit link)*** | | |  | ***Normal error distribution (identity link)*** | | |
| --- | --- | --- | --- | --- | --- | --- | --- | --- | --- |
|  |  |  | ***df*** | ***$\chi2$*** | ***p-value*** |  | ***df*** | ***$\chi2$*** | ***p-value*** |
| Immobility | Intercept | Individual | n.a | n.a | n.a |  | 1 | 0.799 | 0.371 |

p-values < 0.05 are in bold

Model did not converge for the Binomial part of the model.
